# Characterization and Modeling of Nonfilamentary Ta/TaO_x_/TiO_2_/Ti Analog Synaptic Device

**DOI:** 10.1038/srep10150

**Published:** 2015-05-08

**Authors:** Yu-Fen Wang, Yen-Chuan Lin, I-Ting Wang, Tzu-Ping Lin, Tuo-Hung Hou

**Affiliations:** 1 Department of Electronics Engineering and Institute of Electronics, National Chiao Tung University, Hsinchu, Taiwan

## Abstract

A two-terminal analog synaptic device that precisely emulates biological synaptic features is expected to be a critical component for future hardware-based neuromorphic computing. Typical synaptic devices based on filamentary resistive switching face severe limitations on the implementation of concurrent inhibitory and excitatory synapses with low conductance and state fluctuation. For overcoming these limitations, we propose a Ta/TaO_x_/TiO_2_/Ti device with superior analog synaptic features. A physical simulation based on the homogeneous (nonfilamentary) barrier modulation induced by oxygen ion migration accurately reproduces various DC and AC evolutions of synaptic states, including the spike-timing-dependent plasticity and paired-pulse facilitation. Furthermore, a physics-based compact model for facilitating circuit-level design is proposed on the basis of the general definition of memristor devices. This comprehensive experimental and theoretical study of the promising electronic synapse can facilitate realizing large-scale neuromorphic systems.

Energy-efficient, fault-tolerant, massively parallel neuromorphic computing 1 is a computing paradigm that is superior to conventional computers in adaptively processing unstructured and imprecise sensory inputs and performing tasks, such as pattern and voice recognition, robot control, and navigation. Among various implementation technologies, synaptic electronics is an emerging technology aimed at facilitating neuromorphic computing through the use of bio-inspired hardware systems 2. The main device component in synaptic electronics is a low-power, analog electronic synapse, the so-called memristor 3,4, which emulates both the functionality and the two-terminal structure of a biological synapse. A resistive random access memory (RRAM)-based synapse 5,6,7,8,9,10,11,12,13,14 exhibits excellent scaling potential beyond 10 nm 15, a compact 4F^2^ cell size, full CMOS compatibility, ultralow energy consumption per synaptic operation on the order of picojoules 8, and capability of realizing three-dimensional (3D) neural network 9. These features outperform those of other electronic synapse candidates based on phase-change memory 16,17, ferroelectric memory 18, and floating-gate devices 19. Most RRAM-based synaptic devices utilize the filamentary conduction mechanism 20, which leads to the following limitations: (1) Many filamentary synaptic devices exhibit gradual RESET but abrupt SET characteristics 5,6,7,8,9. Implementing concurrent inhibitory and excitatory synapses is not possible, which may hinder the full potential of neuromorphic computing. (2) Because of the filamentary conduction, reducing the synaptic conductance in aggressively scaled devices is difficult. High conductance poses challenges in reducing power consumption, and device characteristics are more susceptible to parasitic line resistance in high-density neural networks. Furthermore, the area overhead of leaky integrate-and-fire neuron circuits becomes appreciable because a larger capacitor is required to integrate the high current. (3) The multiple conductance states of filamentary synaptic devices are controlled by an extremely low number of defects in the nanoscale filament 21. Therefore, these devices are subjected to large conductance fluctuations because of the intrinsic randomness of defect numbers, especially in the low conductance regime 8,9,10,11. Although the neuromorphic system is fault-tolerant, a high degree of conductance fluctuations still adversely affects computational accuracy 9. For overcoming these limitations, new studies have explored RRAM-based synaptic devices that utilize nonfilamentary (homogeneous) switching mechanisms 12,13,14. However, exotic materials, such as praseodymium calcium manganese oxide (PCMO) 12,13, and processes incompatible with semiconductor fabrication, such as sol-gel 14, are often required for fabricating these devices, thus posing additional challenges for high-density integration. Furthermore, the device characteristics and operating mechanism of such nonfilamentary RRAM-based synaptic devices are considerably less understood compared with those of filamentary RRAM-based synaptic devices.

Recently, a Ta/TaO_x_/TiO_2_/Ti device has shown great potential for implementing high-density crossbar memory arrays with numerous highly desirable features including extremely high endurance, forming-free, self-compliance, self-rectification, multiple-level-per-cell capability, and a semiconductor-friendly material and fabrication process 22,23. The resistance change in this device is determined by homogeneous barrier modulation (HBM) induced by oxygen ion migration 23. In this paper, we report an experimental and theoretical study of analog synaptic features in the Ta/TaO_x_/TiO_2_/Ti device. Because of its nonfilamentary mechanism, this device overcomes the limitations of conventional filamentary synaptic devices and shows promising properties for neuromorphic computing, including concurrent inhibitory and excitatory synaptic plasticity, and low synaptic conductance with minimal fluctuation. We present a physical model of HBM that quantitatively describes both the steady-state (DC) and dynamic (AC) evolution of synaptic states. Furthermore, we propose an analytical compact model based on the physical model of HBM and the general definition of memristor devices to facilitate circuit-level simulations in future large-scale neuromorphic system designs.

## Results

**Figure 1:**
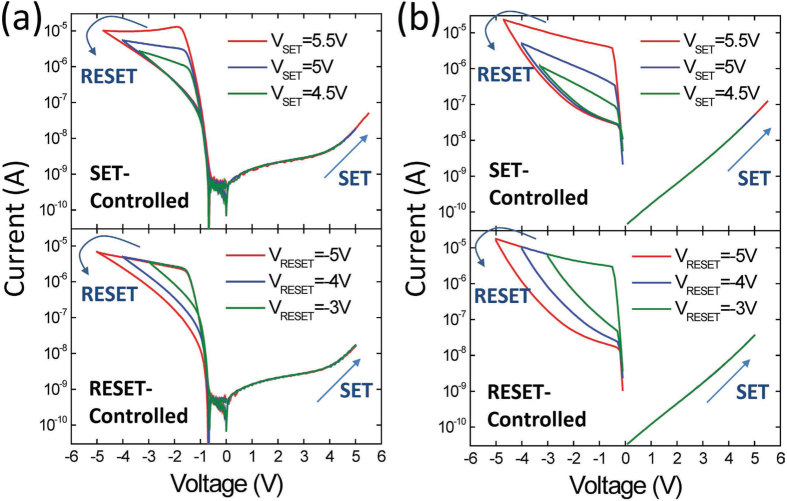
Multiple resistance states by DC voltage sweep and simulation results. (**a**) Typical bipolar resistive-switching *I*-*V* curves of the Ta/TaO_x_/TiO_2_/Ti device. Multiple resistance states were controlled by employing a gradual SET process in which various SET voltages were used (SET-controlled mode) and a gradual RESET process in which various RESET voltages were used (RESET-controlled mode). The conditions for the SET-controlled mode were *V*_SET_ = 5.5 V, *V*_RESET_ = −4.75 V; *V*_SET_ = 5 V, *V*_RESET_ = −4 V; *V*_SET_ = 4.5 V, *V*_RESET_ = −3.35 V. The conditions for the RESET-controlled mode were *V*_SET_ = 5 V, *V*_RESET_ = −5 V; *V*_SET_ = 5 V, *V*_RESET_ = −4 V; *V*_SET_ = 5 V, *V*_RESET_ = −3 V. (**b**) Simulation of SET-controlled and RESET-controlled multiple resistance states using the HBM model. The main device features were successfully reproduced.

**Figure 2:**
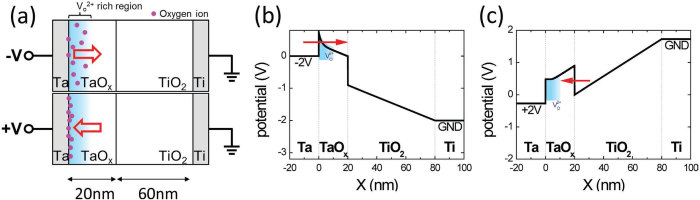
O^2−^ migration and homogeneous barrier modulation model. (**a**) Schematic diagrams showing O^2−^ migration in the V_O_-rich (oxygen-deficient) layer near the Ta electrode during bipolar SET and RESET operations. Calculated band diagrams of low resistance state (after SET) at (**b**) −2 V read and (**c**) +2 V read. At −2 V read, the effective barrier width of electron tunneling from Ta was modulated by considering the charge distribution near the Ta/TaO_x_ interface. At +2 V read, the higher TaO_x_/TiO_2_ barrier suppressed electron tunneling into TaO_x_, and the barrier modulation effect at the Ta/TaO_x_ interface had a negligible effect.

The studied Ta/TaO_x_/TiO_2_/Ti device (see Methods Section) exhibits multiple resistance states that are adjustable by either the SET strength or RESET strength. Figure 1a shows typical current-voltage (*I*-*V*) curves of multiple resistance states, which can be precisely controlled by appropriate SET and RESET voltages. The operating conditions were chosen to show that an identical high-resistance-state (HRS) can be set to different low-resistance states (LRS’s) by using different SET strengths and that, similarly, an identical LRS can be reset to different HRS’s by using different RESET strengths. Because of the rectifying *I*-*V* characteristics, the resistance states can be read out only at a negative voltage, and the SET transition is less apparent. An effective SET process requires at least +4 V (see Figure S1 in Supplementary Information). The HBM-based switching mechanism illustrated in Fig. 2 is supported by experimental findings that TaO_x_ is the main resistive-switching layer and the tunnel barrier to homogeneous current transport 23. Because of the strong oxygen scavenging capability of Ta, an oxygen vacancy (V_O_)-rich TaO_x_ region containing a substantial number of nonlattice oxygen ions (O^2−^) exists near the Ta electrode (the blue region in Fig. 2a). For simplicity, negatively charged O^2−^ is assumed to migrate easily under local electric field *F* in V_O_-rich TaO_x_, and V_O_ are treated as immobile shallow donor-like dopants. During RESET at a negative voltage, O^2−^ migrates toward the bulk of the V_O_-rich TaO_x_ region, thereby increasing the effective barrier width of electron tunneling from Ta into TaO_x_ and decreasing the device conductance at a negative read voltage. During SET at a positive voltage, O^2−^ migrates toward the interface of Ta and TaO_x_, thereby decreasing the effective barrier width of electron tunneling and increasing the device conductance at a negative read voltage. Furthermore, the barrier modulation effect at the Ta/TaO_x_ interface has little influence on the electron tunnel barrier between TiO_2_ and TaO_x_, and a high TaO_x_/TiO_2_ barrier considerably suppresses the current at a positive read voltage, resulting in the characteristic rectifying *I*-*V* curve of the Ta/TaO_x_/TiO_2_/Ti device.

A one-dimensional (1D) simulation was performed to validate the proposed HBM model. O^2−^ migration was described using the diffusion and drift components of O^2−^ hopping through local potential wells 24,25. The O^2−^ density (*N*_*O*_) is obtained using the continuity equation as follows:


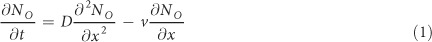


The diffusion coefficient is obtained using *D* = 1/2 *a*^*2*^ *f*. exp(-*E*_*a*_*/kT*), and the drift velocity is expressed as *v* = *a f*. exp(-*E*_*a*_*/kT*). sinh(-*q* γ *a F/kT*), where *a* (0.05 nm) is the effective hopping distance and also the mesh size of numerical calculation 23,26, *f* (10^13^ Hz) is the attempt-to-escape frequency 23,26, *kT* (T = 300 K) is the thermal energy, *q* is the elementary charge, γ is a fitting parameter used to account for the field dependence, and *F* is the electric field; *E*_*a*_ is the activation energy of migration, and it is assumed to be 0.65 eV 23 in the region where the V_O_ density (*N*_*V*_) is greater than 2 × 10^20^ cm^−3^, and to be 2 eV in the region where *N*_*V*_ is lower than 2 × 10^20^ cm^−3^. It is well known that oxygen diffusion is much faster in nonstoichiometric metal oxides than that in stoichiometric oxides because of the high concentration of oxygen defects 27,28, and this fact is considered using this simplifying assumption on *E*_*a*_. More discussions on the choice of simulation parameters can be found in Supplementary Information. *N*_*V*_ is assumed using an exponentially decaying function, with the highest *N*_*V*_ being at the Ta/TaO_x_ interface, which agrees with the gradient substoichiometric TaO_x_ composition observed experimentally 23. The immobile shallow donor-like V_O_ is positively charged (V_O_^2+^) at nonzero *F*. The Poisson equation is solved for the entire Ta/TaO_x_/TiO_2_/Ti stack by considering the effect of both *N*_*O*_ and V_O_^2+^density (*N*_*V+*_) as:


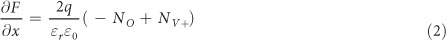


where ε_r_ is the dielectric constant of TaO_x_ or TiO_2_ and ε_0_ is the permittivity of vacuum. After obtaining the potential profile across the device stack, the tunnel current through the TaO_x_ barrier is calculated using the Wentzel-Kramers-Brillouin (WKB) approximation and considering the field-dependent barrier lowering and the thermionic emission component. Figure 1b displays the DC *I*-*V* curves of SET-controlled and RESET-controlled multiple resistance states, obtained from the simulation; the curves show reasonable agreement with the experiments. The discrepancies in the calculated current in the high resistance state and the rectifying condition at a positive bias are attributed to defect-assisted transport processes other than tunneling in TaO_x_.

**Figure 3:**
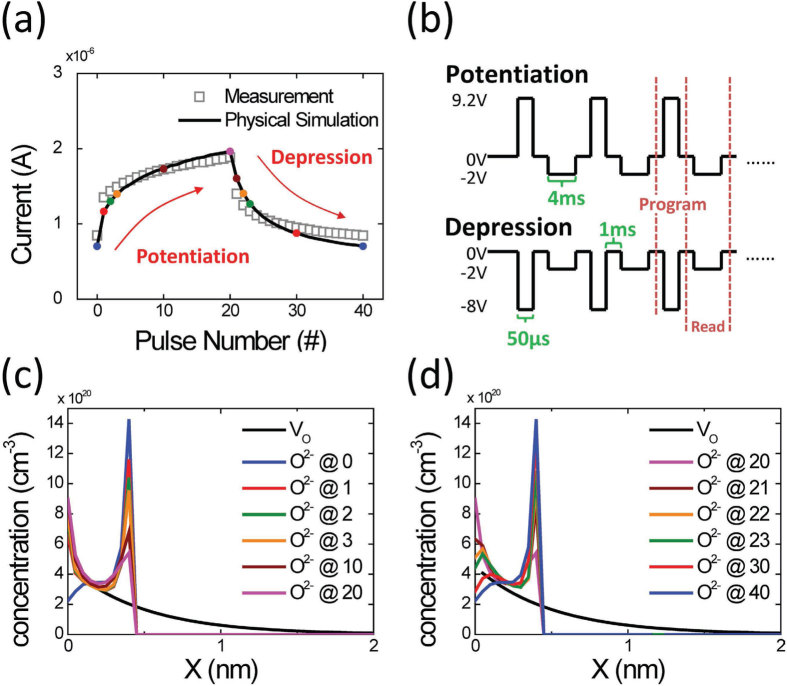
Potentiation and depression by applying AC pulse. (**a**) Measured and simulated potentiating (1^st^ pulse to 20^th^ pulse) and depressing (21^st^ pulse to 40^th^ pulse) curves of the Ta/TaO_x_/TiO_2_/Ti device, obtained by applying AC pulses successively. (**b**) The AC pulse waveforms used for potentiation and depression. A read pulse of −2 V was applied to measure the cell conductance after every potentiating and depressing pulse. All time intervals between read or write pulses were 1 ms. The evolution of O^2−^ concentration near the Ta/TaO_x_ interface is shown for (**c**) the 1^st^ potentiating pulse to the 20^th^ potentiating pulse and (**d**) the 21^st^ depressing pulse to the 40^th^ depressing pulse. Charge accumulation at the Ta/TaO_x_ interface and at the low-*E*_*a*_ TaO_x_/high-*E*_*a*_ TaO_x_ interface reduced the internal electric field and suppressed further O^2−^ migration, resulting in the conductance change saturating during potentiation and depression.

The gradual transition among multiple resistance states induced by the SET and RESET strengths can be applied to implement concurrent inhibitory and excitatory synapses where the synaptic conductance can be increased and decreased using potentiating and depressing AC pulses, respectively. Increasing the number of pulses progressively is equivalent to programming the cell at a higher voltage because of the voltage and time dependence of ion migration 29. Figure 3a,b show the measured potentiating and depressing curves using consecutive AC pulses and the corresponding pulse waveforms. A read pulse of −2 V was applied to measure the cell conductance after every potentiating and depressing pulse. The 1D simulation result showed excellent agreement with the AC pulse measurement, validating the HBM model under both steady-state and transient conditions. The conductance change is not linearly proportional to the pulse number. The first few potentiating or depressing pulses exert considerably strong effects, and the change in the cell conductance gradually saturates. The nonlinearity in the potentiating and depressing curves is similar to those reported in other RRAM-based synapses 8,9,10,11,12,13,14, and it can be explained by the evolution of O^2−^ during potentiation and depression, as shown in Fig. 3c,d. O^2−^ migration occurs easily in the low-*E*_*a*_ region with a high V_O_ concentration. At the first potentiating pulse, O^2−^ moves rapidly from the TaO_x_ bulk toward the Ta/TaO_x_ interface. The conductance change saturates because charge accumulation reduces the internal electric field and suppresses further O^2−^ migration toward the interface. A similar phenomenon can also be cited to explain the saturation of the depression curve. Furthermore, in contrast to the large and random pulse-to-pulse conductance fluctuations in typical filamentary synapses 8,9,10,11, the conductance in the Ta/TaO_x_/TiO_2_/Ti device shows smooth and monotonic transitions between consecutive pulses, which is desirable for improving the learning accuracy in neuromorphic systems. The negligible pulse fluctuation is attributed to the higher number of ions involving in the state change.

**Figure 4:**
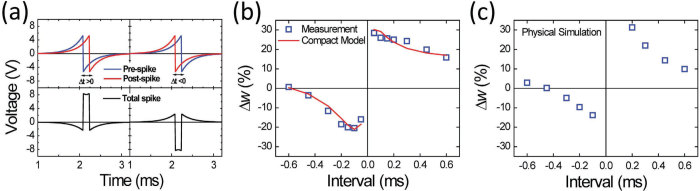
Spike-timing-dependent plasticity (STDP). (**a**) Biomorphic-action-potential-like waveforms used for STDP measurement and simulation. The pre-spike pulse was applied to the Ta electrode, and the post-spike pulse was applied to the Ti electrode. The potentiation and depression actions were controlled by the relative timing of the pre- and post-spikes (*Δt*). The synapse weight change (*Δw*) was positive for *Δt* > 0, whereas it was negative for *Δt* < 0. (**b**) The measured *Δw* as a function of *Δt* for the Ta/TaO_x_/TiO_2_/Ti device. The red line shows the fitting result of using the physics-based compact model. (**c**) Simulation results for STDP, obtained by considering oxygen ion migration and the homogeneous barrier modulation model.

The synaptic weight *w* in a biological neural network is modulated using the spike-timing-dependent plasticity (STDP), and it is typically measured according to the cell conductance. Instead of the square pulses shown in Fig. 3b, biological STDP is triggered by the relative timing of pre- and post-synaptic spikes 30. STDP facilitates the Hebbian learning rule 31; therefore, it is a critical function for learning and memory. The STDP characteristics of the Ta/TaO_x_/TiO_2_/Ti device were measured using a biomorphic-action-potential-like waveform (Fig. 4a), which was generated using:


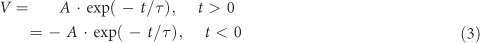


where *A* is the amplitude and *τ* is the decay constant. The pre-spike was sent to the Ti electrode and the post-spike was sent to the Ta electrode; the time difference between the spikes is denoted by *Δt*. Figure 4b,c show the measurement and simulation of the STDP synapse weight change *Δw*, which is defined as the percentage change in the cell conductance after the STDP event. If the pre-spike precedes the post-spike (*Δt* > 0), the polarity of the net spike is mostly positive, resulting in the potentiating action. If the post-spike precedes the pre-spike (*Δt* < 0), the polarity of the net spike is mostly negative, resulting in the depressing action. Furthermore, as *Δt* decreases, the amplitude of the net spike increases, leading to larger *Δw*.

**Figure 5:**
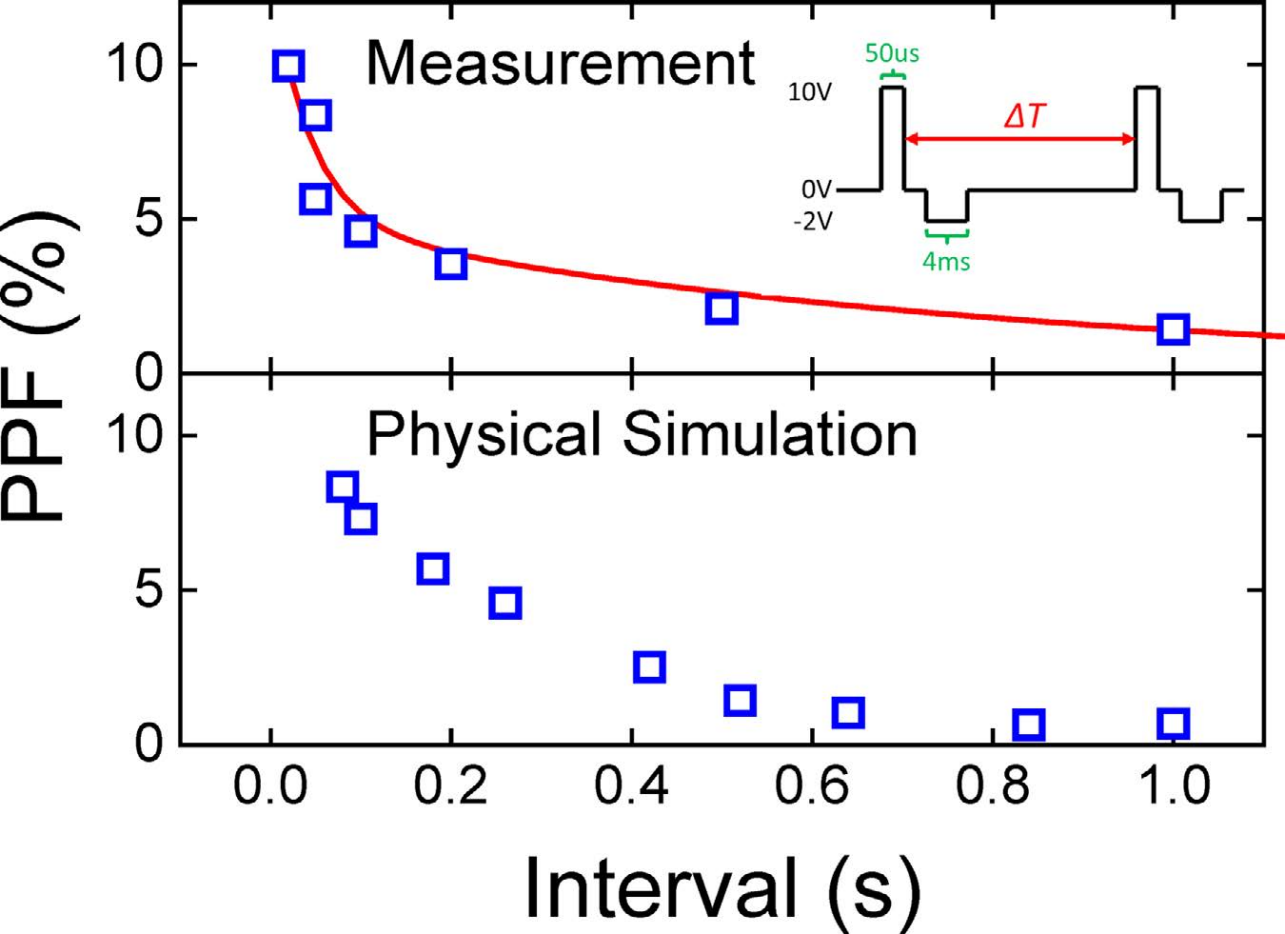
Paired-pulse facilitation (PPF). The inset shows the waveform used for the PPF measurement and simulation. Two identical potentiating pulses, each followed by a negative read pulse, were separated by different time intervals. The PPF ratio is defined as the incremental percentage change in the conductance readouts after the first and second pulses. Both measured and simulated PPF ratios are shown as a function of the time interval between the potentiating pulses. The red line shows the empirical fitting result of using Eq. (4). The two fitting time constants, τ_1_ (45 ms) and τ_2_ (800 ms) correspond to the fast and slow decaying terms, respectively, and agree with time scales of biological synapses 32.

Paired-pulse facilitation (PPF) in biological synapses involves temporal summation on inputs, *i.e.* reducing the time interval between two sequential potentiating pulses enhances synaptic weight 32. This function provides additional flexibility in processing information in the frequency domain. Figure 5 shows the pulse waveform and measurement results for PPF in the Ta/TaO_x_/TiO_2_/Ti device. The PPF ratio is defined by:


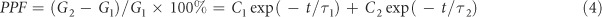


where *G*_*1*_ and *G*_*2*_ are the conductance readouts after the first and second pulses, respectively. The two fitting time constants, τ_1_ (45 ms) and τ_2_ (800 ms) correspond to the fast and slow decaying terms, respectively. These time scales agree with those of biological synapses 32. The PPF effect can also be modeled using the HBM model in which O^2−^ diffuses from the Ta/TaO_x_ interface toward the TaO_x_ bulk during the time interval between pulses. The simulation results in Fig. 5 also agree with experimental data. The longer the time interval is, the weaker the memory effect of the previous pulse on the consecutive pulse.

## Discussion

**Figure 6:**
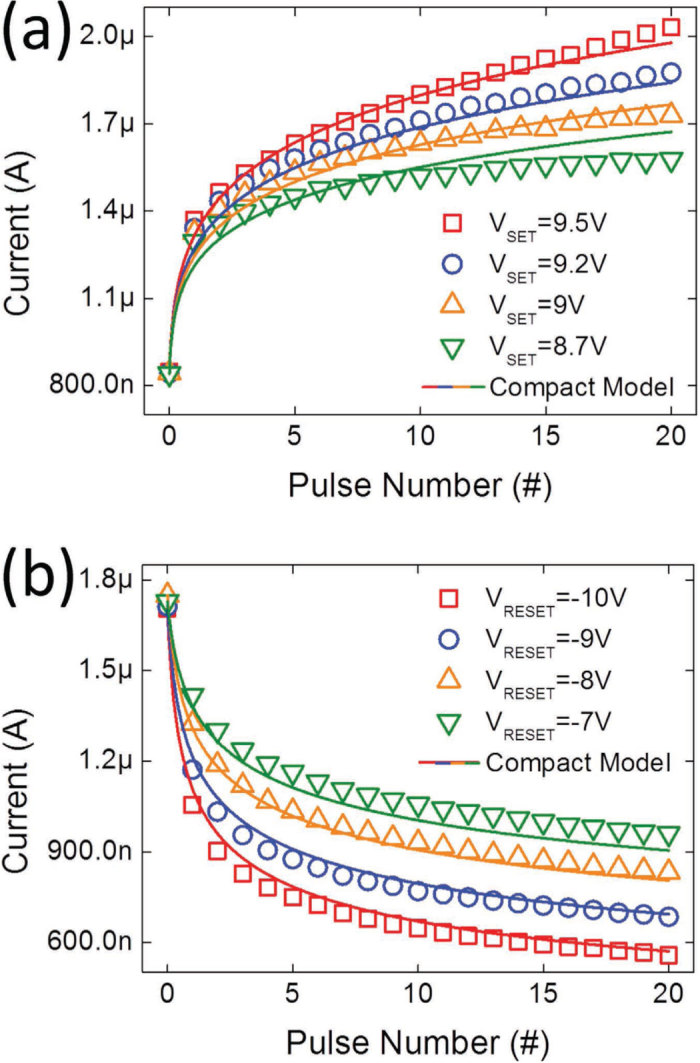
Physics-based compact model fitting. (**a**) Potentiating and (**b**) depressing curves of the Ta/TaO_x_/TiO_2_/Ti device for four pulse amplitudes. The measured data (symbols) and the compact model fitting (lines) are in excellent agreement.

The *I*-*V* characteristics of general memristive devices are described using the following two coupled equations 3:


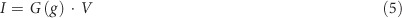



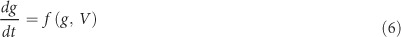


where *g* is an internal state variable. The conductance of memristive devices is determined according to its internal state variable, and the change rate of the internal state variable is determined according to its present state and the applied voltage. On the basis of oxygen ion migration and the HBM model for the Ta/TaO_x_/TiO_2_/Ti device, a physics-based compact model that complies with the general definition of memristive devices was developed to enable large-scale simulation of neuromorphic systems. The compact model is expressed as:


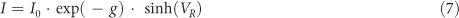



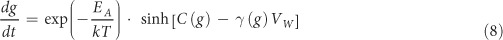


where *V*_*R*_ and *V*_*W*_ are the read voltage and the potentiating/depressing voltage, respectively. Equation (7) describes the tunnel current through TaO_x_ during the read operation using a constant *I*_0_ and an effective tunnel gap *g*. Equation (8) describes the change in *g* resulting from O^2−^ migration through drift and diffusion processes during potentiation and depression. The velocity of O^2−^ migration is influenced by the local O^2−^ density and the electric field in the present state, which can be obtained only by numerically solving Eqs. (1) and (2) simultaneously. The analytical model represented by Eq. (8) uses two linear fitting functions of *g*, field-independent function *C*(*g*) and field-acceleration function γ (*g*), rather than two constants, to account for the state-dependent velocity of O^2−^ migration. Different *C*(*g*) *and* γ (*g*) were used for potentiation and depression because the charge distributions and field-acceleration process are different for forward and backward O^2−^ migration. Figure 6a,b show that the compact model fits the potentiating and depressing curves with high accuracy at four voltages. The fitting accuracy can be increased using higher-order fitting polynomials for *C* (*g*) *and* γ (*g*). Furthermore, this compact model can be applied to simulate STDP characteristics, as shown in Fig. 4b. Because STDP is triggered using a waveform with a time-varying amplitude (See Fig. 4a), the favorable agreement between the measurement and simulation results demonstrates that this compact model is applicable over a wide range of voltages and times.

In summary, the synaptic features of the Ta/TaO_x_/TiO_2_/Ti device were investigated experimentally and theoretically, for demonstrating the feasibility of the device as a building block in future hardware-based neuromorphic computing systems. The device can be used for implementing concurrent inhibitory and excitatory synapses, and it provides superior performance compared with the typical filamentary synaptic devices in reducing synaptic conductance and state fluctuation. The potentiating and depressing evolution of synaptic states for DC and AC inputs, STDP, and PPF can be satisfactorily explained using the HBM model involving O^2−^ migration. Furthermore, because of the improved physical understanding of the operating mechanism, a compact model based on the general definition of memristive devices accurately describes the measurement results obtained over a wide range of voltages and times.

## Methods

The Ta/TaO_x_/TiO_2_/Ti cell was fabricated using a shadow-mask process and had a device area of 10^4^ μm^2^. The bottom and top electrodes were formed by depositing 100-nm-thick Ti and 100-nm thick Ta layers through DC magnetron sputtering, respectively. To prepare the bilayer TaO_x_/TiO_2_ at the bottom electrode, 60-nm-thick TiO_2_ and 20-nm-thick TaO_x_ layers were sequentially deposited through reactive DC magnetron sputtering of metal targets at room temperature in an Ar-O_2_ atmosphere. DC electrical characteristics were measured using an Agilent B1500A semiconductor analyzer in a voltage-sweep mode. Pulse measurements were performed using an Agilent B1530A waveform generator/fast measurement unit.

## Author Contributions

Y.F.W., T.P.L., and T.H.H. performed the 1D numerical simulation. Y.C.L. and I.T.W. performed electrical measurements and constructed the compact model. I.T.W. fabricated the samples. Y.F.W. and T.H.H. co-wrote the paper. All authors discussed the results and commented on the manuscript.

## Additional Information

**How to cite this article**: Wang, Y.-F. *et al.* Characterization and Modeling of Nonfilamentary Ta/TaO_x_/TiO_2_/Ti Analog Synaptic Device. *Sci. Rep.* **5**, 10150; doi: 10.1038/srep10150 (2015).

## Supplementary Material

Supplementary Information
