# Supplementary material for: Characterization and Modeling of Nonfilamentary Ta/TaOx/TiO2/Ti Analog Synaptic Device
Source: Sci Rep. 2015 May 8;5:10150. doi: 10.1038/srep10150 (PMC4424833; doi:10.1038/srep10150)
Supplement: Supplementary Information [file srep10150-s1.pdf]

Supplemental Information

Characterization and Modeling of Nonfilamentary $Ta/TaO_x/TiO_2/Ti$

Analog Synaptic Device

Yu-Fen Wang, Yen-Chuan Lin, I-Ting Wang, Tzu-Ping Lin, and Tuo-Hung Hou

DC SET Transition: The SET transition in the $Ta/TaO_x/TiO_2/Ti$ device is gradual, which is different from the abrupt SET typically observed in the filamentary RRAM. The DC SET transition is further complicated by the self-rectifying characteristics, which hinder the direct measurement of SET transition at the positive bias. Similar to the SET-controlled MLC shown in Figure 1, the gradual DC SET transition can be characterized by investigating the dependence of the SET strength and the resulted LRS at a negative bias from the same HRS. Figure S1 shows that an effective SET process requires at least $V_{SET} > +4$ V.

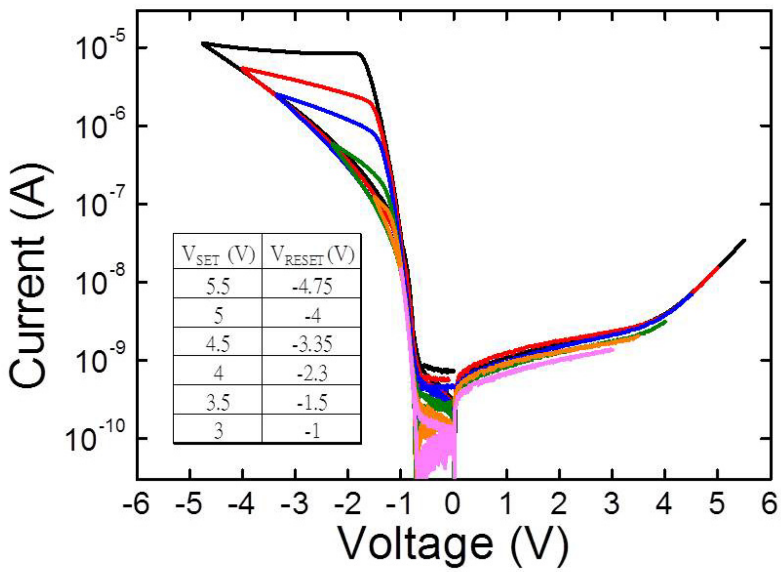

Figure S1. SET transition. Typical multiple resistance states of the $Ta/TaO_x/TiO_2/Ti$ device controlled by a gradual SET process by using various SET voltages. An effective SET process that modulates the resistance readout at negative voltages requires at least +4 V.

Simulation Parameters E_a and *a*: The E_a value of 0.65 eV in the high V_o region was determined by the activation energy of the retention measurement in Ref. [23]. The choice of E_a=2 eV in the low V_o region reflects the fact that oxygen diffusion is much faster in nonstoichiometric metal oxides than that in stoichiometric oxides because of the high concentration of oxygen defects. The oxygen diffusion data in TaO_x or Ta_2O_5 is not well established in the literature. In an oxygen-rich amorphous TaO_x (x=3-7), E_a was estimated about 1.2 ± 0.1 eV [S1]. In ZrO_2 and TiO_2, E_a for volume diffusion was measured as 2 eV to 2.5 eV [S2]. With a high V_o concentration in doped ZrO_2, E_a can reduce to as low as 0.89 eV. In our simulation, as long as E_a is high enough to restrict the oxygen drift/diffusion mainly occurring within a short range near the Ta/TaO_x interface (high V_o region), we have verified that the E_a values, as low as 1.2 eV, do not significantly affect the simulation results. For the same reason, the choice for the abrupt instead of continuous change on E_a simplifies the fitting efforts, but does not affect the simulation conclusions.

Regarding the choice of a = 0.05 nm, a is the effective hopping distance and also the mesh size of numerical calculation. To improve the numerical convergence and spatial resolution, similar to that in Ref. 23 and 26, a smaller a was used whereas a = 1 nm has been used in Ref. 24 and 25. Fig. S2 shows both are mathematically equivalent when used with caution.

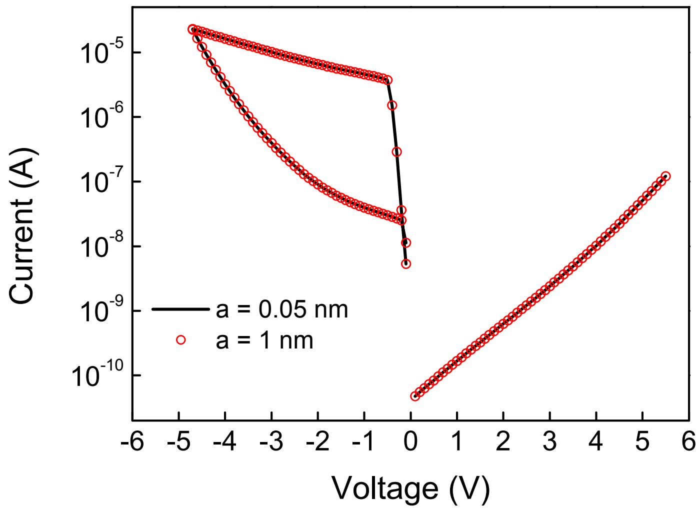

Figure S2. Simulation of DC voltage sweep using different *a* values. The simulation results are independent of the value of the effective hopping distance a, which is also the mesh size used in the numerical calculation.

Supporting Information References

[S1] Nakamura, R. *et al.* Diffusion of oxygen in amorphous Al_2O_3, Ta_2O_5, and Nb_2O_5. *J. Appl. Phys.* **116**, 033504 (2014).

[S2] Brossmanna, U., Wurschumb, R., Sodervall, U., & Schaefer, H.-E. Oxygen diffusion in ultrafine grained monoclinic ZrO_2. *J. Appl. Phys.* **85**, 7626-7654 (1999).
